# IP-10 Is a Potential Biomarker of Cystic Fibrosis Acute Pulmonary Exacerbations

**DOI:** 10.1371/journal.pone.0072398

**Published:** 2013-08-16

**Authors:** George M. Solomon, Carla Frederick, Shaoyan Zhang, Amit Gaggar, Tom Harris, Bradford A. Woodworth, Chad Steele, Steven M. Rowe

**Affiliations:** 1 Department of Medicine, Division of Pulmonary Sciences and Critical Care Medicine, University of Colorado Denver, Denver, Colorado, United States of America; 2 Lung and Cystic Fibrosis Center, Women and Children’s Hospital of Buffalo, Buffalo, New York, United States of America; 3 Department of Medicine, University of Alabama at Birmingham, Birmingham, Alabama, United States of America; 4 Department of Pediatrics, University of Alabama at Birmingham, Birmingham, Alabama, United States of America; 5 Department of Cell, Integrative, and Developmental Biology, University of Alabama at Birmingham, Birmingham, Alabama, United States of America; 6 University of Alabama at Birmingham Lung Health Center, University of Alabama at Birmingham, Birmingham, Alabama, United States of America; 7 Department of Surgery, Otolaryngology, University of Alabama at Birmingham, Birmingham, Alabama, United States of America; 8 Cystic Fibrosis Research Center, University of Alabama at Birmingham, Birmingham, Alabama, United States of America; National Jewish Health, United States of America

## Abstract

**Background:**

Cystic fibrosis (CF) is characterized by acute pulmonary exacerbations (APE). The CF nasal airway exhibits a similar ion transport defect as the lung, and colonization, infection, and inflammation within the nasal passages are common among CF patients. Nasal lavage fluid (NLF) is a minimally invasive means to collect upper airway samples.

**Methods:**

We collected NLF at the onset and resolution of CF APE and compared a 27-plex cytokine profile to stable CF outpatients and normal controls. We also tested IP-10 levels in the bronchoalveolar lavage fluid (BALF) of CF patients. Well-differentiated murine sinonasal monolayers were exposed to bacterial stimulus, and IP-10 levels were measured to test epithelial secretion.

**Results:**

Subjects hospitalized for APE had elevated IP-10 (2582 pg/mL [95% CL of mean: 818,8165], N=13) which significantly decreased (647 pg/mL [357,1174], P<0.05, N =13) following antimicrobial therapy. Stable CF outpatients exhibited intermediately elevated levels (680 pg/mL [281,1644], N=13) that were less than CF inpatients upon admission (P=0.056) but not significantly different than normal controls (342 pg/mL [110,1061]; P=0.3, N=10). IP-10 was significantly increased in CF BALF (2673 pg/mL [1306,5458], N=10) compared to healthy post-lung transplant patients (8.4 pg/mL [0.03,2172], N=5, P<0.001). IP-10 levels from well-differentiated CF murine nasal epithelial monolayers exposed to *Pseudomonas* PAO-1 bacteria-free prep or LPS (100 nM) apically for 24 hours were significantly elevated (1159 ± 147, P<0.001 for PAO-1; 1373 ± 191, P<0.001 for LPS vs. 305 ± 68 for vehicle controls). Human sino-nasal epithelial cells derived from CF patients had a similar response to LPS (34% increase, P<0.05, N=6).

**Conclusions:**

IP-10 is elevated in the nasal lavage of CF patients with APE and responds to antimicrobial therapy. IP-10 is induced by airway epithelia following stimulation with bacterial pathogens in a murine model. Additional research regarding IP-10 as a potential biomarker is warranted.

## Introduction

Cystic Fibrosis (CF) is an autosomal recessive genetic disorder resulting from mutations of the Cystic Fibrosis Transmembrane Conductance Regulator (CFTR) gene [1]. Derangements of the epithelial anion channel results in epithelial dysfunction, predominantly affecting the lungs, pancreas, gastrointestinal system, and reproductive tract. Pulmonary morbidity dominates the disease, and results in acute and chronic pulmonary infections [2]. Much of the morbidity in CF is due to periods of acute decline of lung function with worsening inflammatory and infectious burden in the lungs, known as acute pulmonary exacerbations (APE). Recurrent APEs are associated with the progressive decline in FEV_1_ that characterizes progressive CF lung disease, and ultimately leads to bronchiectasis and end organ dysfunction [3].

Chronic rhinosinusitis is thought to contribute to the pathogenesis of CF airways disease by the unified airway hypothesis [4] The sinus epithelium closely resembles the ion transport and mucociliary clearance properties of the lung, and the pathogenesis of the onset and progression of chronic sinus disease is thought to be very similar to lower airway pathology [5] Like the lower respiratory tract, the sinus airways become chronically infected, which provides a nidus for recurrent pulmonary contamination [6]. Furthermore, exacerbations of chronic rhinosinusitis, in part due to viral infections, may precede and herald the descending pulmonary infectious and inflammatory state that results in APE [5] As such, sinus airway pathology has been proposed to be a significant contributor to the onset of chronic bacterial infection in the lower respiratory tract [7] and may contribute to the progression of lung disease by inciting CF pulmonary exacerbations [5,8,9] and act as a reservoir for descending lower respiratory tract infections.

Nasal lavage fluid (NLF) is readily obtained biologic fluid that has been previously used to monitor inflammation of the upper airway, and may also reflect disease activity in the CF lung [10]. This minimally invasive method allows for characterization of airway inflammatory markers, and is much less invasive than bronchoalveolar lavage, which is not performed routinely and is associated with substantive risk. Prior studies involving the collection of nasal lavage in stable CF patients have implicated the role of potent pro-inflammatory cytokines including IL-8 [11], but a thorough characterization of this biological fluid has not been performed to date in CF, or in the setting of APE. New cytokine multiplex platforms can provide a ready means for rapidly evaluating the cytokine profile of biologic fluids, delivering a new means to assess NLF for a greater variety of potential inflammatory mediators that may contribute to respiratory decline or herald its onset. Our study sought to characterize the upper airway inflammation in CF patients, including in the setting of APE. We collected NLF from CF patients upon diagnosis of APE and after completing a treatment course, in comparison to individuals who were clinically stable. We identified elevated levels of interferon γ-induced protein 10 kDa (IP-10), also known as CXCL10, in the NLF of patients during APE that was dynamic and quite sensitive to routine APE care. *In vitro* studies indicated IP-10 is rapidly and robustly expressed by airway epithelia upon exposure to bacterial pathogens, supporting results in the clinic and suggesting that IP-10 may serve as a sensitive and dynamic biomarker of APE in CF, and possibly other airway diseases.

## Methods

### Human Subjects

Written informed consent was obtained from all subjects and study protocol was approved by the University of Alabama, Birmingham Institutional Review Board for Human Use. All Subjects were provided opportunity to read and ask questions on the consent form before participation as approved by the University of Alabama, Birmingham Institutional Review Board for Human Use. In the acute exacerbation cohort, CF patients diagnosed by the treating physician for acute pulmonary exacerbation who are treated at UAB Hospital or the Children’s Hospital of Alabama were eligible for enrollment. Initial samples were obtained within 48 hrs of admission, and post-therapy samples were obtained within 24 hrs of discharge. In addition, CF patients who were clinically stable and within 5% of baseline lung function were eligible upon presentation to routine outpatient clinic visits. Non-smoking, healthy subjects without known sinopulmonary disease were enrolled as healthy controls for comparison.

For confirmatory studies, remnant bronchoalveolar lavage fluid (BALF) from ten patients with CF who were at baseline lung function who underwent research bronchoscopy as well as five healthy post-lung transplant patients and twenty subjects with non-CF lung disease were also obtained for analysis from an IRB-approved biospecimen repository.

### Total Nasal Symptom Score (TNSS)

At the time of enrollment of the study upon admission for APE and again at the time of discharge, subjects were asked to complete a Total Nasal Symptom Score (TNSS) when nasal lavage was performed, as described previously [12].

### Spirometry

At the time of enrollment of the study, CF subjects underwent routine spirometry including FEV_1_ and FVC maneuvers; this was repeated at the time of discharge as part of routine evaluation for response to treatment for APE. All spirometry conformed to 2005 ATS/ERS standards as previously described and the Wang/Hankinson prediction equation was used for analysis [13].

### Nasal Lavage

Collection of nasal secretions was performed as previously described [14,15]. After clearing excess mucous by forceful exsuflation, the subjects extended their necks approximately 30° from the horizontal while in a sitting position. Five milliliters of normal saline (0.9%) was instilled into one nostril while the subjects refrained from breathing or swallowing using a nasal occluding device. After ten seconds, the subjects flexed their necks and the resultant lavage fluid was collected into a sterile vessel, which was stored on ice until the end of the experiment (for a maximum of 2 h until processed). Nasal lavages were centrifuged at 400×*g* for 10 min at 4 °C. Resultant supernatants were portioned and stored at -80 °C for analysis.

### Bronchoalveolar Lavage

Right middle lobe bronchoalveolar lavage was collected and processed within 2 hrs. Bronchoalveolar lavage samples were then centrifuged at 400×*g* for 10 min at 4 °C. Resultant supernatants were portioned and stored at -80 °C for analysis. The BALF Cohort samples were collected from a repository; only diagnosis was available to the investigative team.

### Multiplex Analysis of Cytokine/Chemokine Profile of NLF and BALF

All nasal lavage and BALF samples were analyzed in batch. The cytokine/chemokine levels in NLF and BALF were performed on the Luminex-200 system and the Xmap Platform (Luminex Corporation, Austin, TX). Fluorescence data were analyzed with Xponent software by using standard curves obtained from serial dilutions of standard cytokines mixtures. This human cytokine 27-Plex Panel kit from Bio-Rad (Hercules, CA) to measure serum concentrations of IL-1, IL-1-receptor antagonist (IL-1-Ra), IL-2, IL-4, IL-5, IL-6, IL-7, IL-8, IL-9, IL-10, IL-12, IL-13, IL-15, IL-17, fibroblast growth factor (FGF), eotaxin, granulocyte colony-stimulating factor (G-CSF), granulocyte macrophage colony-stimulating factor (GM-CSF), interferon-c, IP-10, platelet-derived growth factor BB, MCP-1, MIP-1α, MIP-1β, tumor necrosis factor-alpha (TNF-α), RANTES, and vascular endothelial growth factor (VEGF). Multiplex assays were performed according to the manufacturer instructions.

### Murine Sinonasal Epithelial (MNSE) Cell Culture and Pathogen Stimulation

This protocol was approved by the Committee on the Ethics of Animal Experiments of the University of Alabama Birmingham (Permit #: 100808530). Animals were euthanized for nasal tissue harvest in strict accordance with the recommendations in the Guide for the Care and Use of Laboratory Animals of the National Institutes of Health. All efforts were undertaken to minimize suffering.

Murine Sinonasal Epithelial cells (MNSE) were harvested and differentiated at an air-liquid interface previously described [16,17]. Briefly, tissue was harvested and grown on Costar 6.5-mm-diameter permeable filter supports (Corning Life Sciences, Lowell, MA) submerged in culture medium. The media was removed from the surface of the monolayers on day 4 after reaching confluence and the cells fed via the basal chamber. MNSE cultures were genetically identical and derived from C57 CFTR (−/−) knockout mice or (CFTR +/+) littermate controls. Differentiation and ciliogenesis occurred in all cultures within 10 to 14 days.

After differentiation at air-liquid interface, cells were treated with 10mM DTT apically for 10 min, then washed 3 times with PBS. 100µl of 100ng/ml LPS (Lipopolysaccharides from *E coli* 0111:b4, from SIGMA, Cat #: I4391), PAO-1 (20 hour log growth bacteria free-prep derived from culture), or sterile PBS control was added to apical side and basalaterol medium was changed at the same time. Cell medium was collected 24 hours later and kept at -20°C.

Level of IP-10 in the basolateral compartment were then measured using a murine IP-10 ELISA per the manufacturer’s instructions (Abcam, Cambridge, MA) with samples run in triplicate. Absorbance analysis was performed on a Bio-Rad (Hercules, CA) microplate absorbance spectrophotometer. Linear regression analysis of standard solutions and sample concentration calculation was performed on Microplate Manager 5.2.1(Bio-Rad, Hercules, CA) software.

### Human Sinonasal Epithelial (MNSE) Cell Culture and Pathogen Stimulation

Human subjects with and without CF undergoing routine sinus surgery were recruited to obtain remnant surgical specimens. Written informed consent was obtained from all subjects and study protocol was approved by the University of Alabama, Birmingham Institutional Review Board for Human Use. All Subjects were provided opportunity to read and ask questions on the consent form before participation as approved by the University of Alabama, Birmingham Institutional Review Board for Human Use.

Human Sinonasal Epithelial cells (HNSE) were harvested and differentiated at an air-liquid interface as previously described [18]. Briefly, tissue was harvested and grown on Costar 6.5-mm-diameter permeable filter supports (Corning Life Sciences, Lowell, MA) submerged in culture medium. The media was removed from the surface of the monolayers on day 4 after reaching confluence and the cells fed via the basal chamber. HNSE cultures were obtained from CF (F508del homozygous) and non-CF subjects. Differentiation and ciliogenesis occurred in all cultures within 10 to 14 days.

Cells were treated with 10mM DTT apically for 10 min, then washed 3 times with PBS. 100µl of 100ng/ml LPS (Lipopolysaccharides from *E coli* 0111:b4, from SIGMA, Cat #: I4391) or strerile PBS control was added to apical side and basalaterol medium was changed at the same time. Basolateral cell medium was collected 24 hours later and kept at -20°C.

Level of IP-10 was then measured using a human IP-10 ELISA kit obtained from R&D systems (Minneapolis, MN cat# DIP100). ELISA was run followed the manufacturer protocol with samples run in triplicate. Absorbance analysis was performed on a Bio-Rad (Hercules, CA) microplate absorbance spectrophotometer. Linear regression analysis of standard solutions and sample concentration calculation was performed on Microplate Manager 5.2.1(Bio-Rad, Hercules, CA) software.

### Statistical Analysis

Descriptive statistics (mean, SD, and SEM) were compared using paired Student’s *t* test or ANOVA, as appropriate. All statistical tests were two sided and were performed at a 5% significance level (ie, α = 0.05) using Graphpad Prism version 7.0 (La Jolla, CA) and Microsoft Excel (Microsoft, Seattle, WA). All inferential statistics evaluating IP-10 concentration were log-transformed based on non-Gaussian distribution of IP-10 levels in biologic samples. Fischer’s exact test or χ^2^ was used for categorical variable analysis.

## Results

### Subject characteristics

Thirteen patients were enrolled in the acute exacerbation cohort, and fourteen additional stable CF outpatients were included. Ten normal healthy non-smokers were included as controls. The baseline characteristics of the study subjects are detailed in Table 1. The CF inpatient and outpatient subjects demonstrate a similar baseline lung function (FEV_1_% predicted 60 ± 23 vs 60 ± 14, respectively) and similarly broad age distribution (16-32 vs 19-51 years, respectively). Additionally, the 2 groups demonstrated similar rates of microbiological colonization with common CF pathogens including *Pseudomonas aeruginosa, Burkholderia cepacia*, and *Staphylococcus aureus*. The two groups had similar use of common CF chronic CF therapies as detailed in Table 1. The two groups had a similar rate of CF pancreatic insufficiency and sinus disease, although the inpatient cohort had a trend towards a higher rate of CF-related diabetes mellitus (10/13 inpatients vs. 5/14 outpatients, P=NS by χ^2^). In the acute exacerbation cohort, the average length of hospitalization was 16 ± 2 days.

**Table 1:** Study Subject Characteristics.

			CF Inpatients (n=13		CF Outpatients (n=14)		Normal Subjects (n=10)	
Age (y), mean (range)			22 (16-32)		32 (19-51)		32 (22-44)	
Gender (no. female)			6		4		6	
Race (no. Caucasian)			11		14		9	
**Genotype (n)**								
delta F508/delta F508			7		7			
delta F508/G551D			1		2			
delta F508/Q98X			1		0			
delta F508/1717→A			1		0			
delta F508/2184delA			0		1			
delta F508/G549N			0		1			
E60X/621+1 G→T			2		2			
Unknown			2		2			
**Length of Inpatient stay (days, st dev)**			16 (2)					
**Baseline FEV_1_ % (st dev)**			60 (23)					
**Admission FEV_1_ % (st dev)**			46 (22)					
**Discharge FEV_1_ % (st dev)**			54 (27)					
**Admission Total Nasal Symptom Score (st dev)**			3.2 (1.9)		0.5 (1.0)		1.3 (1.2)	
**Discharge Total Nasal Symptom Score (st dev)**			1.9 (2.3)					
**Admission Nasal Physical Exam Score (st dev)**			6.9 (4.0)		3.8 (2.6)		2.3 (1.5)	
**Discharge Nasal Physical Exam Score (st dev)**			7.9 (2.3)					
**Microbiology (no. subjects)**								
*Pseudomonas aeruginosa*			9		11			
*Burkholderia cepacia*			2		2			
MRSA			11		11			
**Controller Medications (no. subjects)**								
Nebulized Tobramycin			11		7			
Azithromycin			11		5			
Nebulized Colistin			6		5			
Dornase alfa			10		9			
Nebulized Hypertonic Saline			1		3			
**Co-morbidities (no. subject)**								
Pancreatic Insufficiency			13		12			
Diabetes Mellitus			10		5			
Sinus Disease			12		10			
Liver Disease			4		0			

### IP-10 is Elevated in NLF during acute pulmonary exacerbation of CF

**Figure 1:**
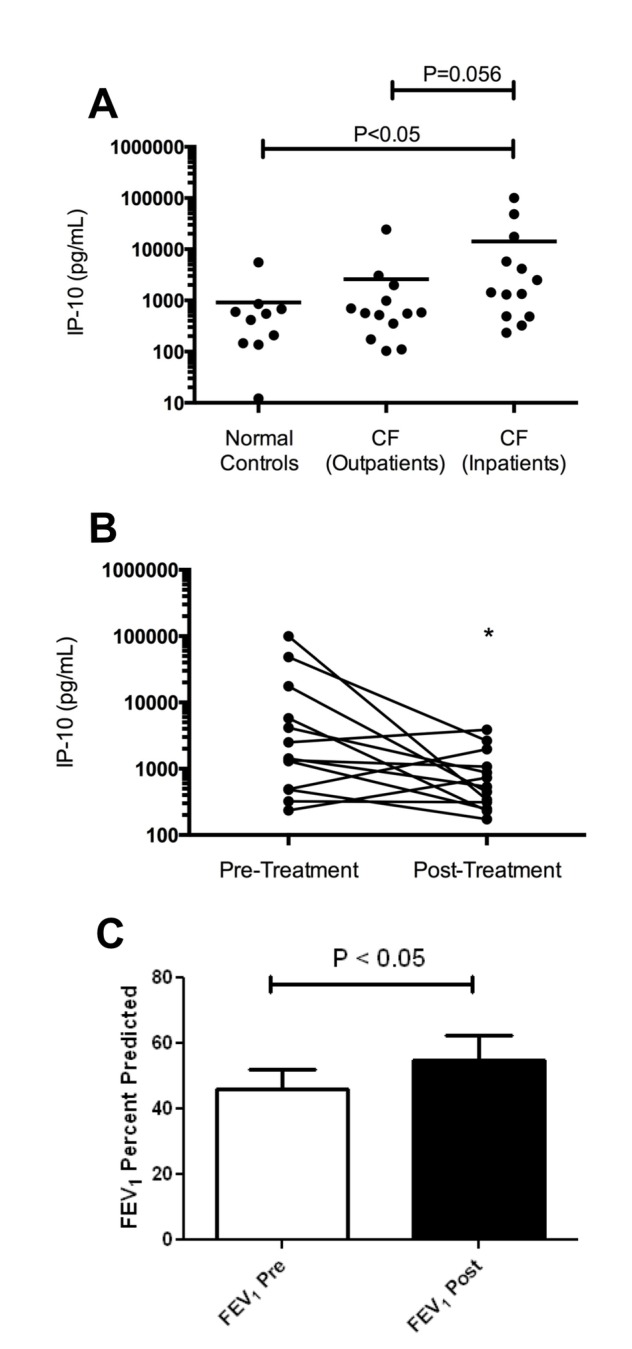
IP-10 levels in nasal lavage fluid. **A**. IP-10 levels in the nasal lavage of CF inpatients hospitalized for APE (N=13) compared to normal (Non-CF) healthy subjects (N=10) and stable CF outpatients (N=13) B. Levels of IP-10 observed in subjects hospitalized and treated for APE (*P<0.05, N=13). **C**. FEV_1_ % predicted before and after treatment for APE in the APE Cohort.

The presence of 27 inflammatory cytokines that have been implicated in lung disease was tested using the Luminex multiplex platform on NLF samples (Table **2**). Of those tested, only IP-10 demonstrated elevated values in the NLF from the inpatient or outpatient subjects. Elevated levels of IP-10 were observed in subjects hospitalized for APE (2582 pg/mL [95% CL of mean: 818, 8165]) compared to normal controls (342 pg/mL [110, 1061]; P<0.05; Figure **1A**). IP-10 levels upon APE diagnosis were also greater than stable CF outpatients (680 pg/mL [281, 1644]) although this did not achieve statistical significance (P=0.056).

**Table 2:** Additional Cytokines Screened in Inpatient CF Patients During Exacerbation.

Cytokine	Pre-Treatment (pg/mL)	Post-Treatment(pg/mL)	P-value
IL-1b	1.48	0.68	0.26
IL-1ra	729.29	518.18	0.46
IL-2	ND	ND	N/A
IL-4	ND	ND	N/A
IL-5	ND	ND	N/A
IL-6	ND	ND	N/A
IL-7	1.71	1.49	0.54
IL-8	62.10	53.37	0.77
IL-9	ND	ND	N/A
IL-10	ND	ND	N/A
IL-12	ND	ND	N/A
IL-13	ND	ND	N/A
IL-15	1.03	0.75	0.21
IL-17	ND	ND	N/A
FGF	ND	ND	N/A
Eotaxin	ND	ND	N/A
G-CSF	8.53	5.99	0.58
GM-CSF	ND	ND	N/A
IFN-γ	ND	ND	N/A
MCP-1	1.35	0.77	0.18
MIP-1a	ND	ND	N/A
MIP-1b	2.49	1.35	0.15
PDGF	ND	ND	N/A
RANTES	ND	ND	N/A
TNF-α	ND	ND	N/A
VEGF	32.14	24.87	0.42

ND = Not Detectable; N/A = not applicable values pre/post-treatment not detectable

Following standard therapy for CF APE that included inpatient IV antibiotics and supportive care, IP-10 significantly and consistently decreased compared to pretreatment values (647 pg/mL [357, 1174], P<0.05, Figure **1B**). Resolution of elevated IP-10 was associated with a trend toward improved total nasal symptom score (TNSS, 3.2 ± 1.9 at admission vs 1.9 ± 2.3 upon discharge, P=0.10). As expected, FEV_1_% predicted improved on APE therapy (46 ± 22 at admission vs. 55 ± 27 upon discharge, P <0.05; Figure **1C**). The change in FEV_1_% predicted did not significantly correlate with changes in IP-10 upon APE treatment (R=0.1, P=0.43).

### IP-10 Levels are elevated in BALF in CF and Disease Controls

**Figure 2:**
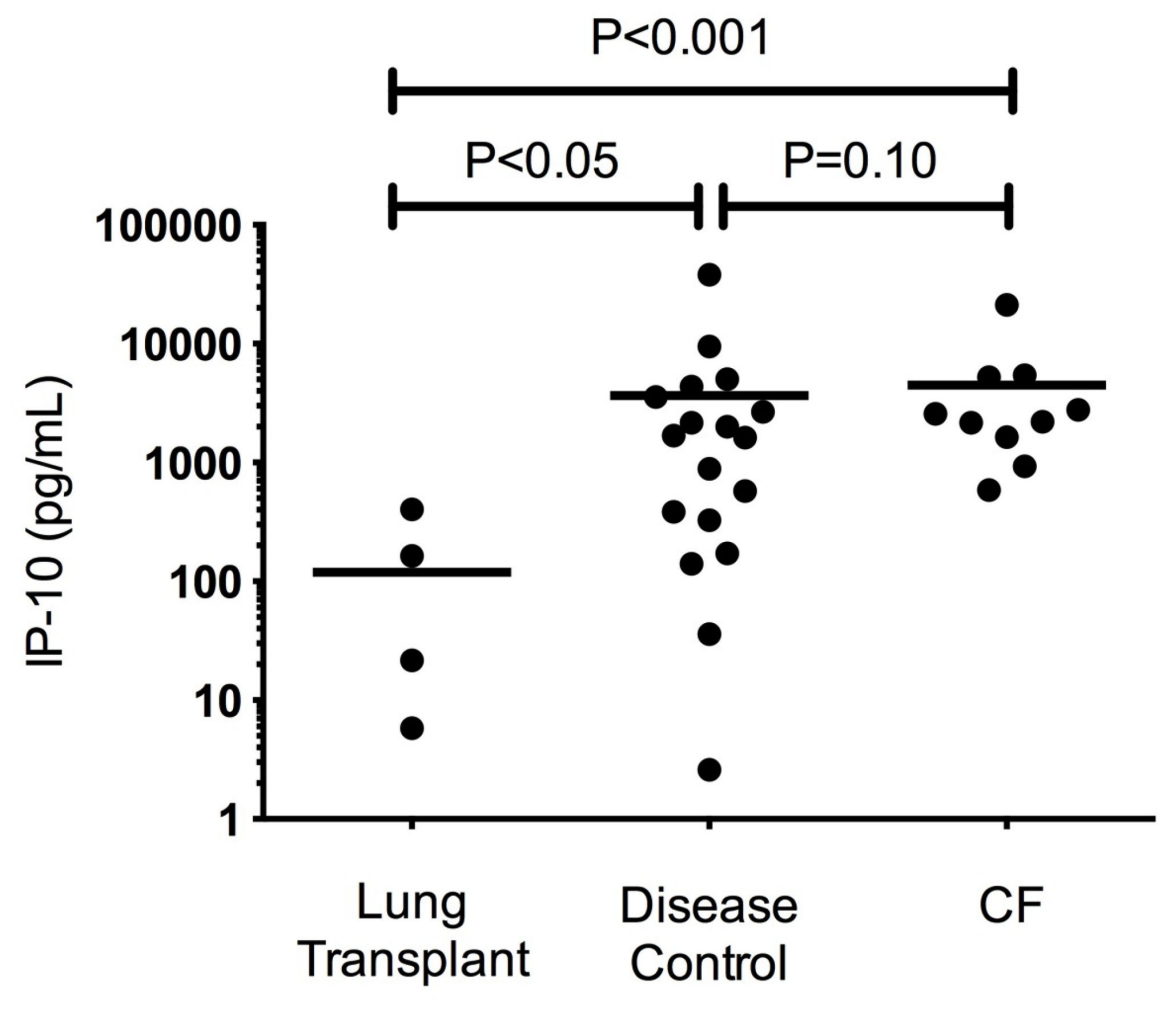
IP-10 levels in bronchoalveolar lavage fluid (BALF). IP-10 levels in the BALF from CF patients (N=10) compared to post-lung transplant controls (N=5) and non-CF lung disease controls (N=20).

To establish whether elevated IP-10 levels in the sinus airway were also reflected in the lower airway, bronchoalveolar fluid from stable CF outpatients were analyzed in comparison to non-CF pulmonary disease controls (which included recurrent pneumonia, cough, and tracheomalacia) and stable post-lung transplant subjects. As shown in Figure **2**, elevated IP-10 was observed in CF BALF (2673 pg/mL [1306, 5458], N=10) which was significantly greater than healthy post-lung transplant patients (8.4 pg/mL [0.03, 2172], N=5, P<0.001). Intermediately elevated IP-10 levels were also observed in the BALF of disease controls (267 pg/mL [36,1968], N=20, P=0.05 post-lung transplant controls), suggesting IP-10 may not be unique to CF APE. Like NLF, BALF did not demonstrate statistically significant difference in the other cytokines tested, suggesting IP-10 may particularly sensitive despite the relatively dilute nature of BALF sampling.

### IP-10 levels increase in a murine model and in human primary epithelial cells after exposure to inflammatory stimuli

**Figure 3:**
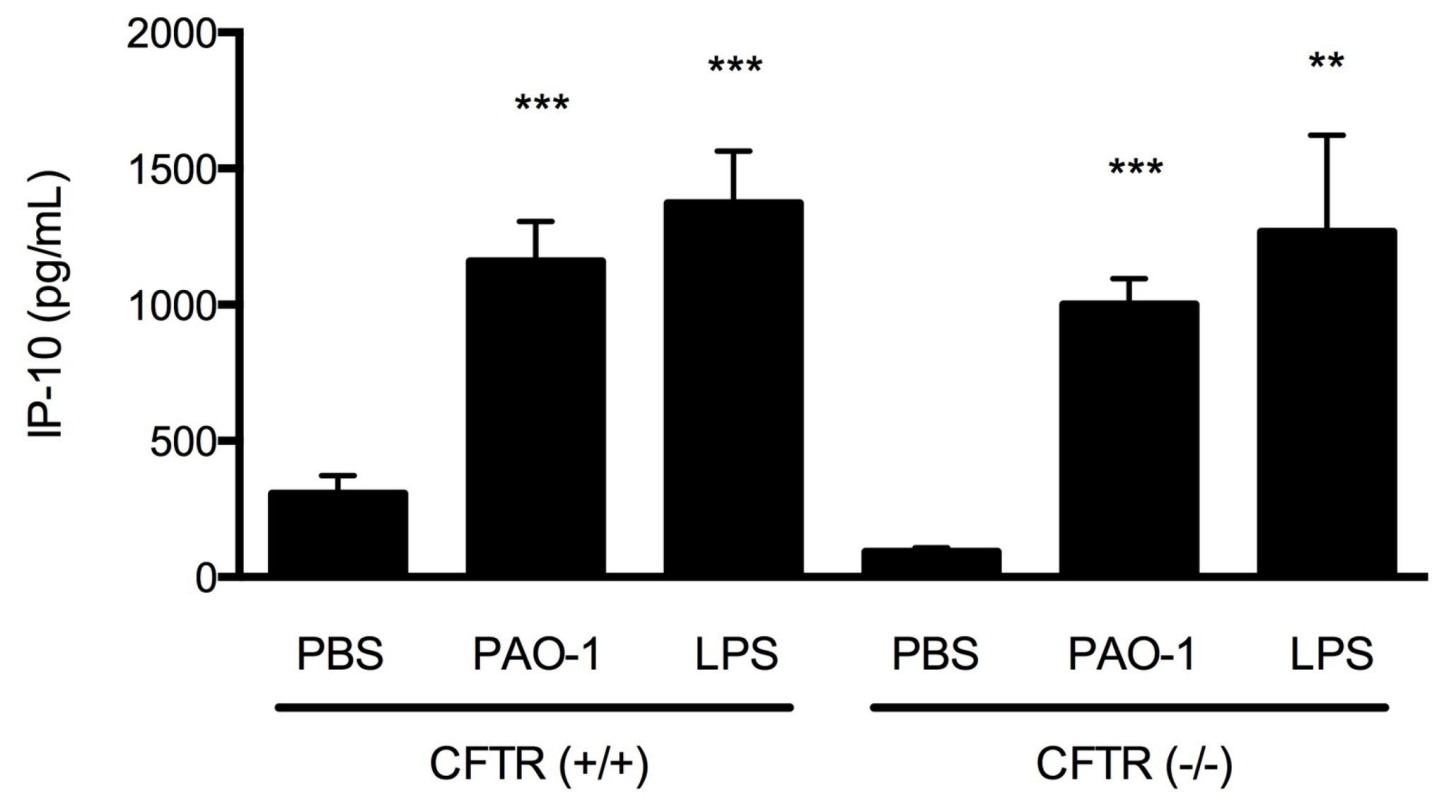
IP-10 levels in the serosal compartment of well-differentiated murine airway epithelial monolayers. IP-10 levels in the basolateral compartment of well-differentiated nasal airway epithelial monolayers derived from CFTR (-/-) and CFTR (+/+) mice exposed to PAO-1 (bacteria-free prep from 20 hour log-phase growth) or sterile LPS (100 nM) apically for 24 hours. No statistically significant difference was observed between CF and WT IP-10 levels at each respective condition. **P<0.005, ***P<0.0001 vs. PBS control; N=10-13 monolayers per treatment.

Previous studies have indicated that IP-10 can be released by respiratory epithelial cells in response to pathologic stimuli such as LPS or PSA antigens [19]. To identify whether this mechanism was operative in the CF airway, and confirm our *in vivo* findings, we evaluated the effect of LPS and PAO-1 on IP-10 levels secreted by well-differentiated primary murine airway epithelial monolayers. As shown in Figure **3**, IP-10 levels in the basolateral compartment of well-differentiated nasal airway epithelial monolayers derived from congenic wild-type (CFTR +/+) mice exposed to PAO-1 (bacteria-free prep from 20 hour log-phase growth) or LPS (100 nm) apically for 24 hours were significantly elevated compared with vehicle treated controls (1159 ± 147 pg/mL, N=13, for PAO-1 vs. 105 ± 26 pg/mL for controls, P<0.001; 1373 ± 191 pg/mL, N=11 for LPS vs. 305 ± 68 pg/mL for controls, N=13, P<0.001), Since the CF genetic defect has been invoked as a potential contributor to the pro-inflammatory environment in humans, and this finding has been recapitulated *in vitro*, we also tested whether IP-10 levels were particularly elevated in CFTR (-/-) sinus airway epithelial cells. IP-10 levels of airway monolayers derived from CF mice (CFTR -/-) were mildly increased at baseline (93 ± 46 pg/mL, N=10), and had a similar increase following immune stimulation (1001 ± 299 pg/mL, N=10, P<0.001 for PAO-1 and 1268 ± 112 pg/mL, N=10, P<0.001 for LPS versus vehicle control specimen; Figure **3**).

**Figure 4:**
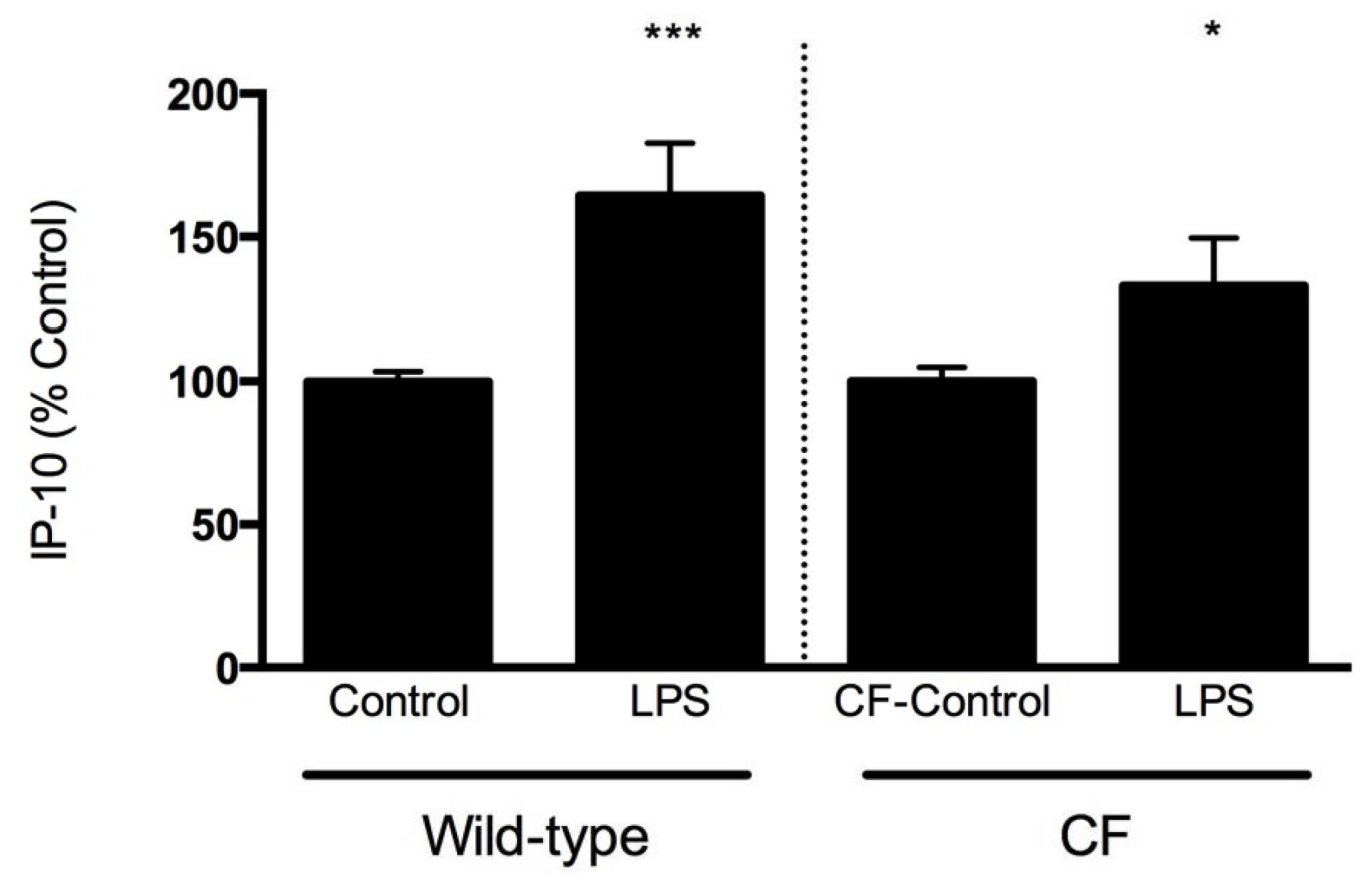
IP-10 levels in the serosal compartment of well differentiated human airway epithelial monolayers. IP-10 levels in the basolateral compartment of well-differentiated nasal epithelial monolayers derived from human subjects with and without CF undergoing routine sinus surgery were exposed to sterile LPS (100nM) apically for 24 hours. *P<0.05, ***P<0.0001; N=6 monolayers per treatment. All data are normalized to baseline (vehicle control) condition to control for variance between donors and cell lots.

To further identify whether this mechanism was operative in the human CF airway, and confirm our *in vivo* findings, we evaluated the effect of LPS on IP-10 levels secreted by well-differentiated primary human sino-nasal epithelial monolayers into the basolateral compartment. To correct for variability between donors at baseline, the data was analyzed as percent of control for each subject (Figure **4**). In the CF-derived cells IP-10 expression increased 33% when stimulated by LPS (100 nM) apically for 24 hours as compared to vehicle control stimulus (P<0.05). A similar increase was observed in cells derived from a non-CF subject; IP-10 expression increased 54% when stimulated by LPS as compared to vehicle control (P<0.0001). Absolute values demonstrated a similar trend. IP-10 levels derived from CF human sinus surgery remnant exposed to LPS were elevated in the CF subject compared to stimulus with vehicle control (31.82 ± 20.56 pg/mL LPS vs. 21.4 ± 9.9 pg/mL vehicle control, N=6 monolayers/condition, P=NS). In the non-CF subject similar stimulation with LPS was observed (205.4 ± 146.0 pg/mL LPS vs. 153.0 ± 135.0 pg/mL controls, N=6 monolayers/condition, P=NS).

## Discussion

Our study demonstrates effective use of nasal lavage fluid (NLF) to define a new potential biomarker in sinopulmonary disease. Previous studies indicate that NLF is feasible and reproducible for defining the inflammatory cell, cytokine, and protein content of the upper airways in children and adults [10]. Pitrez et al. demonstrated that the elaboration of IL-8 in the NLF correlates with simultaneous elaboration in BALF [11]. In contrast, our study was conducted using a screening platform to explore a diverse number of potential cytokines of interest. While our study did not show potent elaboration of IL-8 in NLF, the cytokine was detected as expected in the lower airways. Methodological differences including dilutional techniques between the two studies and our use of a broader screen, which may have limited relative sensitivity to small changes, may explain these differences. Furthermore, in comparison to Pitrez, the present study was conducted in an older group of patients with significantly higher rates of chronic CF complications, which may have impacted detectable differences in IL-8 levels before and after exacerbation, perhaps due to chronically elevated expression. Other studies also indicate that NLF is effective in defining the cytokine and microbiota of the upper airways in CF [11,20]. Despite the recognition of the feasibility of NLF, until now, little prior research to support its use for biomarker discovery or in assessing response to therapeutics has been conducted, and there is no literature that has evaluated the role of NLF in assessing the response to treatment for acute pulmonary exacerbations in CF. Thus our study provides an important contribution, and indicates the need for further research to characterize the role of IP-10 in CF and other sinopulmonary disorders.

The *in vitro* studies in airway epithelial culture and the broad literature regarding the role of IP-10 in acute and chronic infection lend biological plausibility to the findings of our study. IP-10 is a member of the CXC chemokine family, and is released by a wide variety of inflammatory cells and airway epithelia in response to acute inflammation. The activity of this cytokine is mediated by binding to the CXCR3 receptor primarily on T and B lymphocytes, NK cells and macrophages [19] The resultant elaboration of IFN-γ by Th1 cells after homing to the site of inflammation suggests the presence of a potent positive feedback loop for activity in an acute process [21]. This loop system thereby allows for the various downstream effects of this chemokine including induction of apoptosis [22], regulation of bronchial epithelial and type II pneumocyte proliferation and migration [23,24], and regulation of angiostasis [25]. These various effects provide a evidence to suggest a role of IP-10 in pulmonary infectious disease including reduced IP-10 after antibiotic treatment in a murine model of *Mycoplasma pneumoniae* [26], use as a marker of acute tuberculous infection [27], and enhanced susceptibility to *Legionella* *pneumophilia* pneumonia in subjects with impaired IP-10 production [28]. Robust expression of IP-10 in airway cells *in vitro* demonstrates the importance of the surface epithelium, in addition to respiratory inflammatory cells, in initiating and maintaining this inflammatory pathway.

The correlation of IP-10 levels in NLF with BALF in other chronic airway diseases and in airway epithelial culture in the absence of CFTR expression suggests that the elaboration of IP-10 in airways-derived fluid is not specific to CF, although this disorder may be more prone to heightened IP-10 expression, as also suggested by some but not all of the in vitro studies presented here. Given the similarities between inflammatory patterns in CF and other chronic airways diseases [29], the lack of specificity of IP-10 for exacerbations of CF lung disease was not unanticipated. Nevertheless, because the infectious burdens in the CF airway is significantly greater than many other chronic airway diseases, we believe this may lead to a more potent elaboration of IP-10 in CF pulmonary exacerbations and at baseline when compared to other sinopulmonary diseases of similar mechanism; further studies will be needed to explore this hypothesis.”

The murine *in vitro* experiments suggest that high infectious burden, such as that caused by chronic *Pseudomonas aeruginosa* infection or Methicillin resistant *Stapholococcus* *aureus* (MRSA) infection in CF patients, causes a significant response to inflammatory cytokine pathways, as was also observed following exposure to LPS or with the Pseudomonas strain PAO-1 in murine and human sinonasal epithelial monolayers. Of note, the majority of patients (70%) harbored chronic infection with strains of *P. aeruginosa* and were treated for this infection during the APE, which may explain why IP-10 was so readily detected in our NLF population. Further studies will be needed to determine if IP-10 changes are equally sensitive to CF APE treatment in individuals with MRSA infection in the absence of *P. aeruginosa* colonization, or in other populations such as individuals with non-CF chronic rhinosinusitis, since of majority of individuals in our study also harbored MRSA.”

Since IP-10 is significantly elevated in CF, and demonstrates a robust response to treatment of APE, the biomarker may be particularly useful in this disorder. Though correction for multiple comparisons was not performed in our study, confirmatory studies in the lower airways as well as in vitro studies using murine and human epithelial monolayers that indicate IP-10 is robustly expressed in response to bacterial infection indicate IP-10 is elevated in CF APE and may also contribute to the pathobiology of CF pulmonary exacerbations. There is significant interest in defining CF biomarkers that are sensitive to early changes in airway inflammation, and can help identify when APE is resolved, but no current literature identifies a biomarker with this profile [30–34]. Because IP-10 appears to meet many of these characteristics, and can be detected in a relatively simple maneuver that is less invasive than bronchoscopy, IP-10 deserves further consideration as a CF biomarker of interest.

Although our evaluation of BALF and NLF was cross-sectional, our study demonstrates elevated IP-10 in both of these compartments; this is consistent with the unified airways hypothesis and provides additional confidence in the results. The use of sputum as a surrogate for lower airway pathology has also been explored in the literature [35]. Sputum samples have the advantage of being readily accessible and reflecting lower airways sampling, although variability between individuals remains problematic [35]. Recent studies have shown that sputum inflammatory cell and cytokine analysis are both useful in the evaluation of patients at risk for worsening lung disease, especially because a strong negative correlations between IL-8 and neutrophil elastase to percent predicted FEV_1_ has been observed [36,37]. Relatively less is known about the role of sputum biomarkers for diagnosing APE or in response to treatment except the correlation determined by Liou et al of sputum GM-CSF levels and onset of APE [38]. In comparison to NLF, sputum sampling, especially when sputum induction is required, is relatively more difficult, time-consuming, and only available in older children and adults. For this reason, in addition to addressing variability caused by differences in expectorated sputum (vs. intrinsic, non-expectorated mucus), further exploration of NLF as a surrogate for lower airway sampling remains attractive for biomarker development.

While this study demonstrates a potent elaboration of IP-10 amongst a large panel of tested cytokines, it has important limitations. As a single-center evaluation with a relatively small sample size, our findings need to be replicated to determine if this inflammatory cytokine is sensitive for the use as a biomarker for APE. Additional studies to define whether IP-10 levels in NLF are correlated with BALF on a intrasubject basis would further substantiate the use of NLF as a proxy to lower airways inflammation. Larger studies could also help determine whether reduced IP-10 with APE treatment correlates with improvement in clinical parameters, such as FEV_1_, TNSS, or other markers of disease activity, such as CRP. Additional study may also show that rising IP-10 levels herald the onset of APE. Further, evaluating whether IP-10 can be detected in serum could provide an even more convenient fashion to monitor the onset and resolution of APE. Another important limitation of our study is the non-randomized fashion in which it was conducted. As a result, several important differences exist between the inpatient and outpatient CF populations studied. Of note, the CF outpatient population is older and harbors a lower incidence of *Pseudomonas* infection. This Is clinically relevant as patients who are more stable with reduced incidence of CF pulmonary exacerbation are more likely to be older and have reduced incidence of CF complications, including CF-related diabetes. Thereby, while representing a limitation in comparison of disease severity, these differences are clinically relevant and relatively expected given the nature of CF lung disease.

The use of the bioluminex platform was intended in this initial study to screen for potent elaboration of the cytokines in CF APE. The high expression of IP-10 in this setting indicates its relative sensitivity to CF APE. However, it was surprising that additional pro-inflammatory cytokines were not similarly elevated in this population. This may be due to the relatively dilute nature of NLF samples, which could limit detection of other cytokines, particularly when detected in the context of a screening assay.

In summary, we report the first study that characterizes the cytokine profile of NLF in CF, including its response to APE treatment. We demonstrate the elaboration in the airways of the potent inflammatory cytokine IP-10, in part through epithelial expression, and provide evidence that its presence in the NLF may correspond to disease activity, particularly in the presence of *P. aeruginosa and/or* MRSA. Further studies to determine whether IP-10 levels in NLF or serum can serve as biomarkers for the onset and resolution of CF APE, or other disorders dominated by *P. aeruginosa* or other chronic infections are warranted.
